# Altered gut microbiota associated with symptom severity in schizophrenia

**DOI:** 10.7717/peerj.9574

**Published:** 2020-07-29

**Authors:** Shijia Li, Min Zhuo, Xia Huang, Yuanyuan Huang, Jing Zhou, Dongsheng Xiong, Jiahui Li, Ya Liu, Zhilin Pan, Hehua Li, Jun Chen, Xiaobo Li, Zhiming Xiang, Fengchun Wu, Kai Wu

**Affiliations:** 1 School of Biology and Biological Engineering, South China University of Technology, Guangzhou, Guangdong, China; 2 Department of Biomedical Engineering, School of Material Science and Engineering, South China University of Technology, Guangzhou, Guangdong, China; 3 The Affifiliated Brain Hospital of Guangzhou Medical University, Guangzhou Huiai Hospital, Guangzhou, Guangdong, China; 4 Guangdong Engineering Technology Research Center for Translational Medicine of Mental Disorders, Guangzhou, Guangdong, China; 5 Guangdong Engineering Technology Research Center for Diagnosis and Rehabilitation of Dementia, Guangzhou, Guangdong, China; 6 Key Laboratory of Biomedical Engineering of Guangdong Province, South China University of Technology, Guangzhou, Guangdong, China; 7 Department of Biomedical Engineering, New Jersey Institute of Technology, Newark, NY, United States; 8 Department of Radiology, Panyu Central Hospital of Guangzhou, Guangzhou, Guangdong, China; 9 National Engineering Research Center for Tissue Restoration and Reconstruction, South China University of Technology, Guangzhou, Guangdong, China; 10 Department of Nuclear Medicine and Radiology/Institute of Development/Aging and Cancer, Tohoku University, Sendai, Japan

**Keywords:** Schizophrenia, Gut microbiota, Microbiome-Gut-Brain axis, 16S rRNA sequencing, PANSS

## Abstract

**Background:**

The gut microbiome and microbiome-gut-brain (MGB) axis have been receiving increasing attention for their role in the regulation of mental behavior and possible biological basis of psychiatric disorders. With the advance of next-generation sequencing technology, characterization of the gut microbiota in schizophrenia (SZ) patients can provide rich clues for the diagnosis and prevention of SZ.

**Methods:**

In this study, we compared the differences in the fecal microbiota between 82 SZ patients and 80 demographically matched normal controls (NCs) by 16S rRNA sequencing and analyzed the correlations between altered gut microbiota and symptom severity.

**Results:**

The alpha diversity showed no significant differences between the NC and SZ groups, but the beta diversity revealed significant community-level separation in microbiome composition between the two groups (pseudo-*F* =3.337, *p* < 0.001, uncorrected). At the phylum level, relatively more *Actinobacteria* and less *Firmicutes* (*p* < 0.05, FDR corrected) were found in the SZ group. At the genus level, the relative abundances of *Collinsella*, *Lactobacillus*, *Succinivibrio*, *Mogibacterium*, *Corynebacterium*, undefined *Ruminococcus* and undefined *Eubacterium* were significantly increased, whereas the abundances of *Adlercreutzia*, *Anaerostipes*, *Ruminococcus* and *Faecalibacterium* were decreased in the SZ group compared to the NC group (*p* < 0.05, FDR corrected). We performed PICRUSt analysis and found that several metabolic pathways differed significantly between the two groups, including the Polyketide sugar unit biosynthesis, Valine, Leucine and Isoleucine biosynthesis, Pantothenate and CoA biosynthesis, C5-Branched dibasic acid metabolism, Phenylpropanoid biosynthesis, Ascorbate and aldarate metabolism, Nucleotide metabolism and Propanoate metabolism pathways (*p* < 0.05, FDR corrected). Among the SZ group, the abundance of *Succinivibrio* was positively correlated with the total Positive and Negative Syndrome Scale (PANSS) scores (*r* = 0.24, *p* < 0.05, uncorrected) as well as the general PANSS scores (*r* = 0.22, *p* < 0.05, uncorrected); *Corynebacterium* was negatively related to the negative scores of PANSS (*r* = 0.22, *p* < 0.05, uncorrected).

**Conclusions:**

Our findings provided evidence of altered gut microbial composition in SZ group. In addition, we found that *Succinvibrio* and *Corynebacterium* were associated with the severity of symptoms for the first time, which may provide some new biomarkers for the diagnosis of SZ.

## Introduction

Schizophrenia (SZ) is a complex, chronic psychiatric disorder with a heterogeneous genetic and neurobiological background (Bang et al., 2014; Hoekert et al., 2007; McGlashan & Fenton, 1992). Treatments for SZ are available, but their effectiveness is poor for many patients (Higuchi et al., 2019; Kraus et al., 2006). To acquire better therapeutic results, we need to completely understand the pathophysiology of SZ. Previously, researchers have focused on analyzing the human genome (Ripke et al., 2011) and environmental risk factors (Brown et al., 2002; Cannon, Jones & Murray, 2002; Cantor-Graae & Selten, 2005; Mulvany et al., 2001; Sara et al., 2014; Stilo et al., 2017; Van Os, Pedersen & Mortensen, 2004; Varese et al., 2012) to determine the pathogenesis of SZ. However, the identified associations only account for some of the variance in SZ (Wu et al., 2019). Recently, interest in researching the effect of gut microbiota on host physiology and pathology has increased rapidly. The variations in the composition of the gut microbiota influence inflammatory and metabolic pathways across a number of diseases, such as inflammatory bowel disease (Huttenhower, Kostic & Xavier, 2014), obesity and metabolic diseases (Bouter et al., 2017; Hartstra et al., 2015), cancer (Schwabe & Jobin, 2013) and chronic pulmonary diseases (Shukla et al., 2017). Converging evidence also suggests that the gut microbiota communicates with the central nervous system bidirectionally through the microbiome-gut-brain (MGB) axis and thereby influences brain function and behavior (Cryan & Dinan, 2012; Desbonnet et al., 2014; Hsiao et al., 2013; Sampson et al., 2016). A dysregulated MGB axis has been reported in many neuropsychiatric disorders including bipolar disorder (Hu et al., 2019), major depression disorder (Zheng et al., 2016), Alzheimer’s disease (Cattaneo et al., 2017), Parkinson’s disease (Caputi & Giron, 2018) and autism (Tomova et al., 2015).

Recently, a few articles have focused on the role of the MGB axis in SZ. Epidemiological studies have shown that prenatal microbial infections appear to increase the risk of SZ in offspring (Babulas et al., 2006). Additionally, SZ often superinduces gut and digestive disturbances or intestinal inflammation with a high prevalence (Severance et al., 2012, 2015; Sherwin et al., 2016; Yolken et al., 2015). Some clinical studies indicate potential associations between a disturbed gut microbiome and SZ (Lv et al., 2017). Castro-Nallar et al. (2015) sequenced microbes in the oropharynx of patients who have SZ and found a difference between the SZs and normal controls (NCs), which further indicated that the host microbiome have an impact on host’s health. The gut microbiota and its metabolites are critical in promoting neurodevelopment by modulating important agents, such as neurotrophin and neurotransmitters. Fecal microbiota transplantation showed that germ-free mice that received SZ microbiome fecal transplants displayed SZ-relevant behaviors similar to SZ mouse models (Zheng et al., 2019).

Changes in the gut microbiota and its metabolites may cause neuronal damage, apoptosis and abnormal brain development, leading to SZ (Yuan et al., 2019). An increase in intestinal mucosal permeability induced by gut microbiota dysbiosis leads to alterations in intestinal membrane proteins zonulin and mucin and in metabolites indolepropionic acid (IPA), lipopolysaccharides (LPS) and SCFA (Wang, Geier & Howarth, 2016). The metabolite IPA of gut microbiota maintains gut mucosal barrier integrity and Homeostasis of monocytes and T cells (Dodd et al., 2017). The metabolite LPS activates the peripheral immune system, damages the blood brain barrier (BBB), and causes neuroinflammation (Cao et al., 2017); further, LPS and toxic substances are translocated in the gut lumen, aggravating peripheral immune dysfunction to cause neuroimmune activation. The metabolite SCFA protects the brain barrier and intestinal mucosal barrier, and regulates the peripheral immune system and microglia function in the brain and potentially regulates the development and function of meningeal lymphatic vessels in the brain (Louveau et al., 2018), lower levels of SCFA induced by decreased colonic bacteria can damage the intestinal barrier and the BBB, altering microlia vulnerability and morphology and activating immune responses and neuroinflammation. In conclusion, disturbances in the gut microbiota may cause microglia-mediated neuroinflammation and damage to neurons, synapses, and connectivity between brain regions. These disturbances are a possible mechanism for the etiopathology of SZ.

Previous studies discovered that the abundance of some bacteria in the gut of SZs is quite different from that in the gut of NCs. One of these studies found that at the phylum level, *Proteobacteria* was significantly increased in SZs; the genera *Succinivibrio*, *Megasphaera*, *Collinsella*, *Clostridium*, *Klebsiella* and *Methanobrevibacter* were significantly higher, whereas *Blautia*, *Coprococcus* and *Roseburia* were decreased compared to NCs, and receiver operating characteristic curve analysis demonstrated that 12 microbiota could be used to distinguish SZs from NCs (Shen et al., 2018). Another study based on the metagenomic analysis of gut microbiota showed that the numbers of *Lactobacillus* group bacteria were elevated in first-episode SZs and significantly correlated with severity (Schwarz et al., 2018). In addition, the investigation of the gut microbiome in US-based patients with chronic SZ revealed that the phylum *Proteobacteria* was relatively decreased in SZs, and at the genus level, *Anaerococcus* was relatively increased in SZs, while *Haemophilus*, *Sutterella* and *Clostridium* were decreased (Nguyen et al., 2018) and increased negative symptoms were associated with decreased abundance of family *Ruminococcaceae* and greater severity of depressive symptoms was correlated with greater abundance of genus *Bacteroides*. In these studies, the taxonomies of altered bacteria in SZs are inconsistent, and the correlation between altered gut microbiota and symptom severity are not fully understood. The inconsistencies might be due to (1) the small sample size of these studies; (2) various factors, such as region, diet, environment, etc. (Huttenhower et al., 2012); (3) subjects with other mental disorders.

The objective of this study was to characterize the gut microbiome in SZs and preliminary analyze the correlation between the altered gut microbiota and the severity of symptoms. We excluded individuals with any chronic disease that may affect the stability of the gut microbiota, including intestinal inflammation, Constipation, diarrhea and diabetes; expanded the sample size; and controlled the drug use of the NCs to eliminate possible bias. We hypothesized that (1) gut microbial composition might differ between the SZ and NC groups and (2) the altered gut microbiota in SZs might significantly correlate with symptom severity.

## Materials and Methods

### Participants

A total of 162 subjects were collected in this study from September 2017 to February 2019, including 82 SZs and 80 NCs. The SZs were recruited from Guangzhou Huiai Hospital and were diagnosed by trained and experienced clinical psychiatrists according to the structured clinical interview according to the Diagnostic and Statistical Manual of Mental Disorder-IV-Text Revision (DSM-IV-TR) (SCID) criteria (Wu et al., 2018); The psychiatric symptoms were steady >2 weeks; the Positive and Negative Syndrome Scale (PANSS) evaluated the rate of change ≤20% in 2 weeks and the total score of PANSS ≥30. Seventy-five patients were treated with antipsychotics at the time of the study (Supplemental File 1). The exclusion criteria for patients included (1) any other psychiatric Axis I disorder meeting DSM-IV criteria, including schizoaffective disorder, mental retardation, major depressive disorder, bipolar, delirium, dementia, memory disorder and other cognitive disorders; (2) constipation, diarrhea, diabetes, hypertension, heart disease, thyroid diseases or any somatic diseases; (3) a history of epilepsy, except for febrile convulsions; (4) a history of having received electroconvulsive therapy in the past 6 months; (5) lactating, pregnant, or planning to become pregnant; (6) alcohol dependence or (7) noncompliant drug administration or a lack of legal guardians.

Normal controls were recruited in Guangzhou and surrounding areas through multiple methods, including recruitment flyers in the community, internet ads and word-of-mouth. The age, sex and nationality of all NCs were matched with the SZs. The inclusion criteria of NCs were as follows: (1) the Han nationality, no special religious beliefs; (2) 18–65 years; (3) absence of antibiotic intake for the last 3 months and with no diarrhea at present; (4) absence of any chronic disease that may affect the stability of gut microbiota; (5) BMI 18–30 kg/m^2^; (6) absence of any major gastrointestinal tract surgery within 5 years; and (7) absence of any head surgery and no mental disorders.

All participants signed the information consent form, indicating their agreement. The sample collection and the protocol of analysis were approved by Guangzhou Brain Hospital. A questionnaire was conducted among all subjects to collect general information, including age, sex, height, weight, years of education, history of taking medicine, and history of smoking and drinking.

### Fecal sample collection and 16S ribosome RNA sequencing

Fresh fecal samples were obtained from participants, and all of the samples were stored at −80 °C until DNA extraction. A total of 200 mg of each fecal sample was used for DNA extraction.

Community DNA was extracted under the manual of the MOBIO PowerSoil^®^ DNA Isolation Kit 12,888–100 protocol. Prior to sequencing, the DNA was stored in Tris-EDTA buffer solution at −80 °C. To enable amplification of the V4 region of the 16S rRNA gene and add barcode sequences, unique fusion primers were designed based on the universal primer set 515F (5′-GTGYCAGCMGCCGCGGTAA-3′) and 806R (5′-GGACTACNVGGGTWTCTAAT-3′) along with barcode sequences. PCR mixtures in 50 μL reaction volumes contained 1 μL of each forward and reverse primer (10 μM), 1 μL of Easy Pfu DNA Polymerase (2.5 U/μL), 4 μL of dNTPs (2.5 mM), 1 μL of template DNA, 1 μL of double distilled water, and 5 μL of 10 × EasyPfu Buffer. Thermal cycling consisted of an initial denaturation step at 95 °C for 5 min, followed by 30 cycles of denaturation at 94 °C for 30 s, annealing at 60 °C for 30 s, and extension at 72 °C for 40 s, with a final extension step at 72 °C for 4 min. Amplicons from each sample were run on an agarose gel. The expected band size for 515f–806r is approximately 300–350 bp. Amplicons were quantified with Quant-iT PicoGreen dsDNA Assay Kit (cat. no. P11496; ThermoFisher/Invitrogen, Waltham, MA, USA) following the manufacturer’s instructions. According to the manufacturer’s instructions, the amplicon libraries for high-throughput sequencing on the Illumina MiSeq platform were combined in equal amounts and subsequently quantified (KAPA Library Quantification Kit KK4824).

### Bioinformatics and statistical analyses

The raw sequences were processed to concatenate reads into tags according to the overlapping relationship by using QIIME2 (Bolyen et al., 2019). The DADA2 algorithm was performed to demultiplex raw sequences and identify microbial features (Callahan et al., 2016). The output features were rarefied to 1,3581 sequences per sample, which was the lowest value in the dataset. The microbial community structure was characterized using measures of alpha-diversity (within-sample) and beta-diversity (between-samples). The alpha-diversity indices we selected were Evenness, Faith’s Phylogenetic Diversity, Observed Species and Shannon, which represent the evenness and richness of taxa within a single sample, and the differences in diversity between groups were calculated using the nonparametric Kruskal–Wallis *H* test in QIIME2. The beta-diversity indicates differences in taxa composition between groups, which were calculated using Bray-Curtis dissimilarity. Principal coordinate analysis (PCoA) based on the Bray-Curtis distances matrix was used for visualizing sample relationships, and PERMANOVA with 999 permutations was used to assess the statistical significance of beta-diversity distances between groups. Output matrices were ordinated and visualized using the vegan package from R (Oksanen et al., 2019). We used a pretrained Naïve Bayes classifier for taxonomic analysis. This classifier was trained on the Greengenes database (13.8) (DeSantis et al., 2006), and all differential abundances at different taxonomic levels were tested using the Mann–Whitney *U* test. Linear discriminant analysis (LDA) effect size (LEfSe) was used to identify different markers, an alpha = 0.01 was used in the factorial Kruskal–Wallis test among groups, and the log value for the LDA score was set to >2. To determine the association between differential abundance at the genus level and clinical characteristics, we further calculated the residuals of relative abundance of those taxa with significant group differences, controlling for age, sex and years of education, by the ‘vglm’ function in the VGAM package (Yee, 2007). Pearson’s correlations were then calculated between the residuals of relative abundance of those taxa from patients and the PANSS scores. Significances of all tests were set as *p* < 0.05, or FDR corrected *p* < 0.05 (two side). To obtain insight into the possible functional pathways that differ between SZs and NCs, we used PICRUSt (Langille et al., 2013) to calculate contributions of various features to known biological pathways based on KEGG orthology groups (KOs) using the Kyoto Encyclopedia of Genes and Genomes (KEGG) databases (Ogata et al., 2000).

## Results

### Clinical data

A total of 82 SZs and 80 NCs were recruited according to the inclusion criteria. Demographic and clinical characteristics of the groups are presented in Table 1. The SZ and NC groups did not differ in age (*p* = 0.60, uncorrected) or sex (*p* = 0.35, uncorrected). The years of education (*p* = 2.04 × 10^−6^, uncorrected) and BMI (*p* = 0.01, uncorrected) of the SZ group were lower than those of the NC group. The ratio of tobacco using was higher in the SZ group than in the NC group (*p* = 0.01, uncorrected), while alcohol intake was lower (*p* = 3.36 × 10^−8^, uncorrected). Comparing high-density lipoprotein cholesterol (HDL-C), low-density lipoprotein cholesterol (LDL-C) and glucose in serum, the SZ group showed lower values of HDL-C (*p* = 1.43 × 10^−4^, uncorrected), LDL-C (*p* = 7.95 × 10^−6^, uncorrected) and glucose (*p* = 1.38 × 10^−8^, uncorrected) compared to the NC group. In addition, the SZ group showed lower values of total cholesterol (TC) (*p* = 4.95 × 10^−15^, uncorrected) and triglyceride (TG) (*p* = 0.01, uncorrected).

**Table 1:** Demographic characteristic of schizophrenia and normal controls. Values are shown as mean ± SD or ratio.

Characteristic	NC group (*n* = 80)	SZ group (*n* = 82)	*p*-Value
Age	41.03 ± 14.34	42.15 ± 13.13	0.60
Sex (M/F)	39/41	46/36	0.35
BMI (kg/m^2^)*	23.03 ± 3.05	24.48 ± 4.33	0.01
PANSS	–	59.12 ± 18.18	–
Education year	13.95 ± 3.49	11.22 ± 3.51	2.04 × 10^−6^
S-HDL-C (mmol/l)	1.65 ± 0.29	1.40 ± 0.50	1.43 × 10^−4^
S-LDL-C (mmol/l)	3.62 ± 0.95	2.97 ± 0.84	7.95 × 10^−6^
S-Glu (mmol/l)	5.77 ± 1.15	4.83 ± 1.04	1.38 × 10^−8^
TC (mmol/l)	6.24 ± 1.19	4.76 ± 0.94	4.59 × 10^−15^
TG (mmol/l)	1.26 ± 0.69	1.56 ± 0.83	0.01
Tobacco intake (%)	5	20.7	0.01
Alcohol intake (%)	37.5	3.7	3.36 × 10^−8^

**Notes:**

* Eight NCs and 10 SZ patients lacked BMI information.

BMI, body mass index; S-HDL-C, serum high-density lipoprotein cholesterol; S-LDL-C, serum low-density lipoprotein cholesterol; S-Glu, serum glucose; TC, total cholesterol; TG, triglyceride.

### Sequencing data

We obtained 7,456,515 raw sequences from all subjects (*n* = 162), ranging from 15,449 to 95,651. After quality filtering and removal of the chimeric sequences, we obtained 6,817,960 high quality reads for further analysis of bacterial composition, ranging from 13,581 to 90,203 and with a mean of 42,086.2 reads. After clustering all the high-quality reads, a total of 2,031 features were obtained, and the frequency per feature ranged from 2 to 533,200, with a mean of 3,356.9.

**Figure 1:**
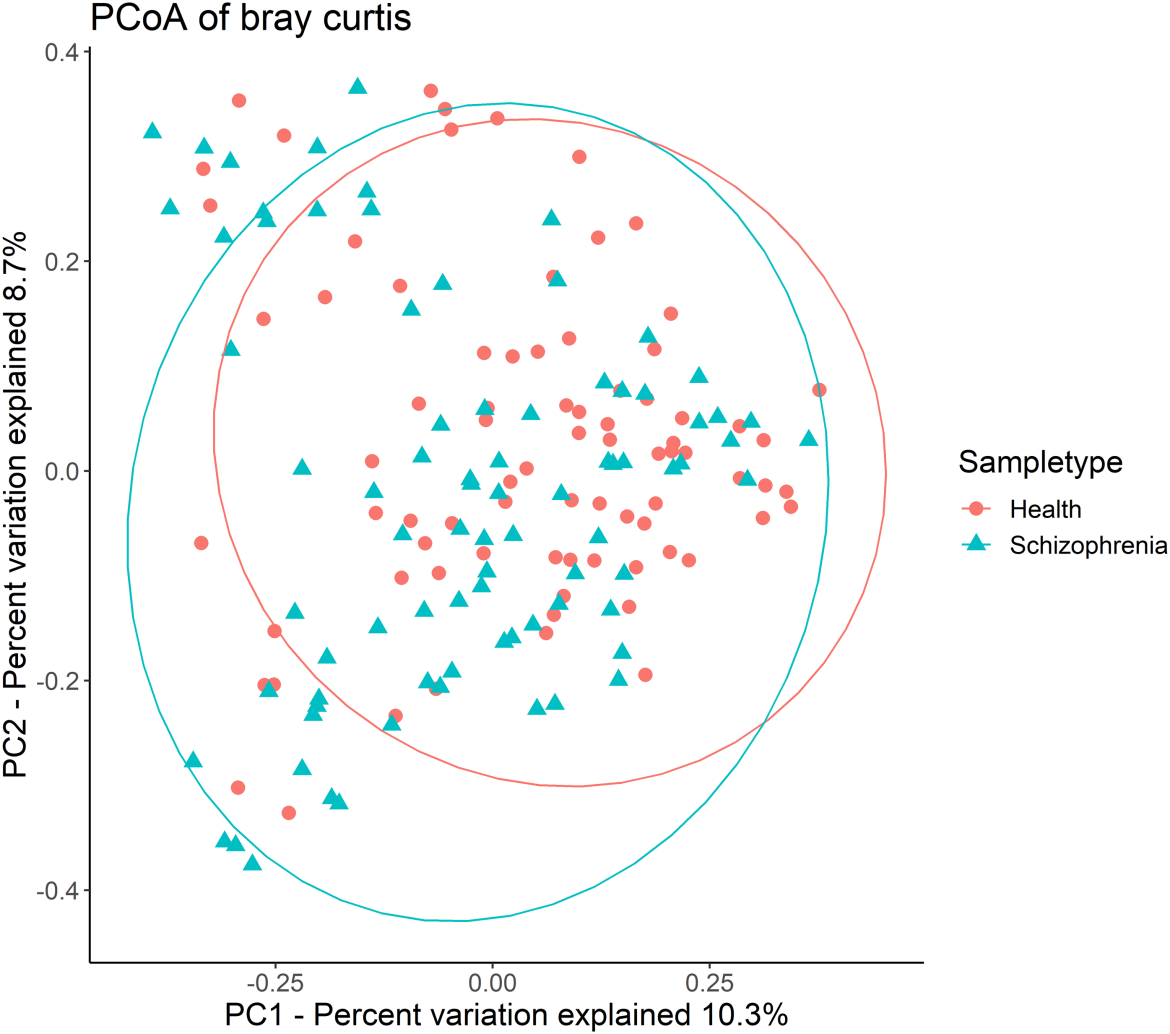
Principal coordinates analysis (PCoA) plot illustrating beta-diversity distance matrices of Bray-Curtis distance comparing sample distributions between the SZ and NC groups. Red dots and green triangles represent NCs and SZ patients, respectively.

Then, alpha-diversity and beta-diversity calculations were performed. The results showed no significant difference in all alpha-diversity indices between the two groups (Table S1). Analysis of beta-diversity indices using Bray-Curtis dissimilarity revealed significant community-level separation between the SZ and NC groups (pseudo-*F* =3.337, *p* = 0.001, uncorrected). PCoA of Bray-Curtis distances showed that the SZ and NC groups formed distinct clusters (Fig. 1). Additionally, the microbiota of the NC group displayed significantly tighter clustering compared to the SZ group, with average Bray-Curtis distances of 0.79 ± 0.05 vs. 0.81 ± 0.06 (*p* = 0.038, uncorrected).

### Bacterial taxonomic compositions and identifications of biomarkers

**Figure 2:**
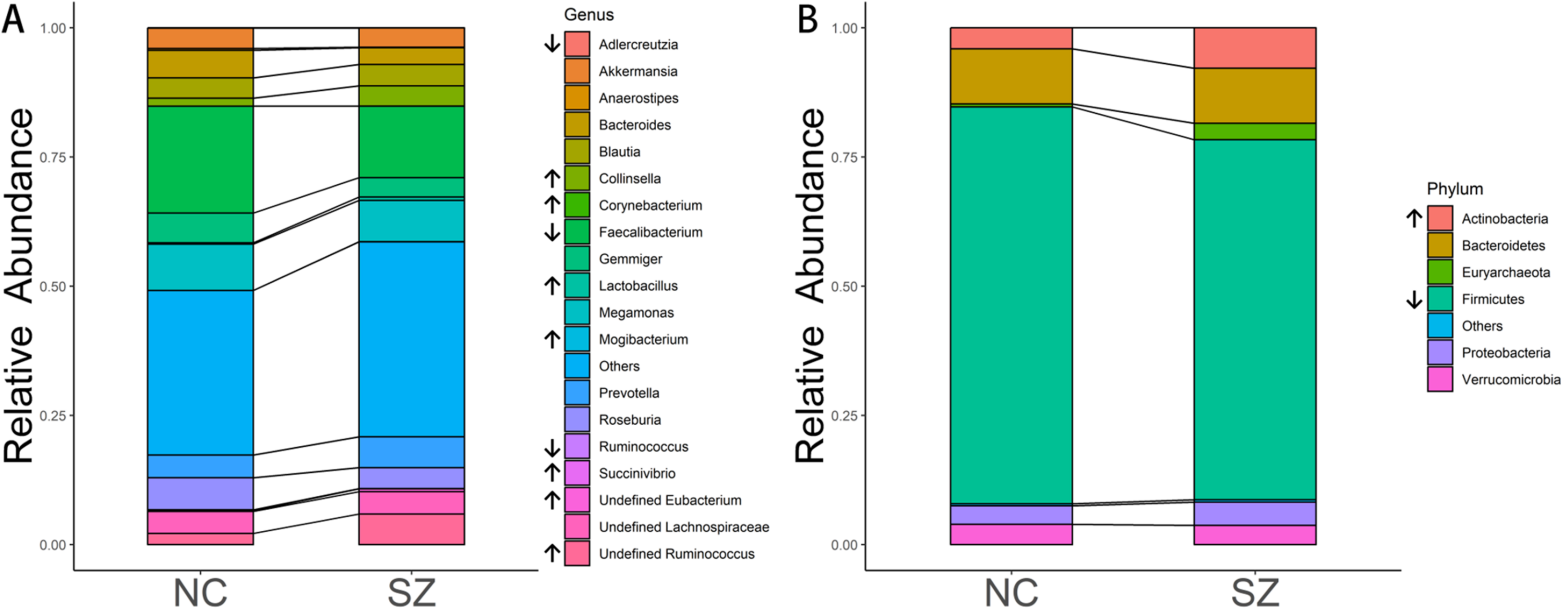
Microbial composition at phylum and genus levels. (A and B) indicate the most abundant genera and phyla in the NC and SZ groups, respectively. Bacteria that were significantly different between the two groups are shown in (A and B) (*p* < 0.05, FDR correction, “↑” represent higher in SZs and “↓” represent lower, respectively).

The predominant bacteria at the phylum level were the same between the SZ and NC groups (Fig. 2B), including *Firmicutes*, *Bacteroidetes*, *Actinobacteria*, *Proteobacteria* and *Verrucomicrobia*. When comparing the relative abundances of the phyla of the two groups, *Actinobacteria* was significantly higher in the SZ group than in the NC group (*p* = 0.0046, FDR corrected), whereas *Firmicutes* was lower (*p* = 0.026, FDR corrected) (Fig. 2B).

At the genus level, the most abundant genus in the SZs was *Faecalibacterium*, followed by *Megamonas*, *Prevotella*, *Ruminococcus* and *Blautia* (Fig. 2A). The bacteria in the NCs were mainly assigned to *Faecalibacterium*, *Megamonas*, *Gemmiger*, *Roseburia* and *Bacteroides*. Genera with different relative abundances between the two groups are shown in Fig. 2A. Compared to the NC group, the relative abundance of undefined *Ruminococcus* (*p* = 0.0052, FDR corrected), *Collinsella* (*p* = 0.00094, FDR corrected), undefined *Eubacterium* (*p* = 8.05 × 10^−6^, FDR corrected), *Lactobacillus* (*p* = 0.0148, FDR corrected), *Succinivibrio* (*p* = 0.0148, FDR corrected), *Mogibacterium* (*p* = 0.0148, FDR corrected) and *Corynebacterium* (*p* = 0.0413, FDR corrected) were significantly higher in the SZ group. However, *Adlercreutzia* (*p* = 0.0148, FDR corrected), *Anaerostipes* (*p* = 0.0025, FDR corrected), *Ruminococcus* (*p* = 0.0083, FDR corrected) and *Faecalibacterium* (*p* = 0.0223, FDR corrected) were higher in the NC group.

**Figure 3:**
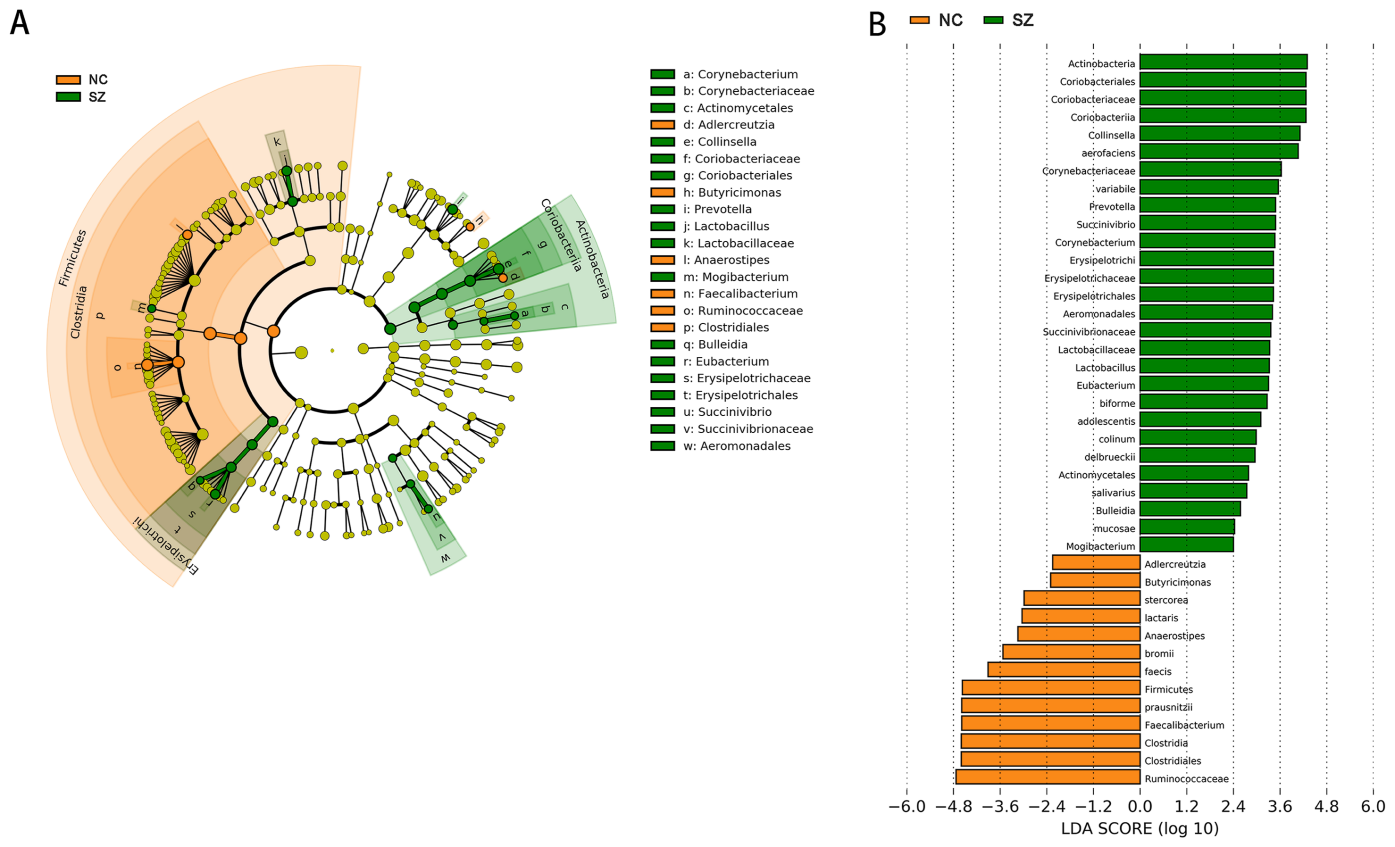
The differently abundant taxa identified using LEfSe analysis. (A) LEfSe cladogram showed the most differentially abundant taxa between the two groups. Taxa enriched for NC in red; SZ enriched taxa in green. The size of each dot is proportional to its effect size. (B) Visualization of only taxa meeting an LDA threshold >2. Taxa with enriched levels in SZs are shown in green, red represented taxa with enriched levels in NCs.

Application of the LefSe method identified a total of 41 features with significantly different abundances between the SZ and NC groups (*p* < 0.01, uncorrected, LDA score > 2) (Fig. 3B). At the phylum level, the NC group was enriched with *Firmicutes*, while *Actinobacteria* was enriched in the SZ group (*p* < 0.01, uncorrected, LDA score > 2). We also observed that the NC group was differentially enriched with the genera *Anaerostipes*, *Faecalibacterium*, *Adlercreutzia*, *Butyricimonas* (*p* < 0.01, uncorrected, LDA score > 2), whereas the SZ group was enriched with *Lactobacillus*, *Mogibacterium*, *Bulleidia*, *Eubacterium*, *Succinivibrio*, *Corynebacterium*, *Collinsella* and *Prevotella* (*p* < 0.01, uncorrected, LDA score > 2) (Fig. 3A).

### Functional properties predicted by PICRUSt

**Figure 4:**
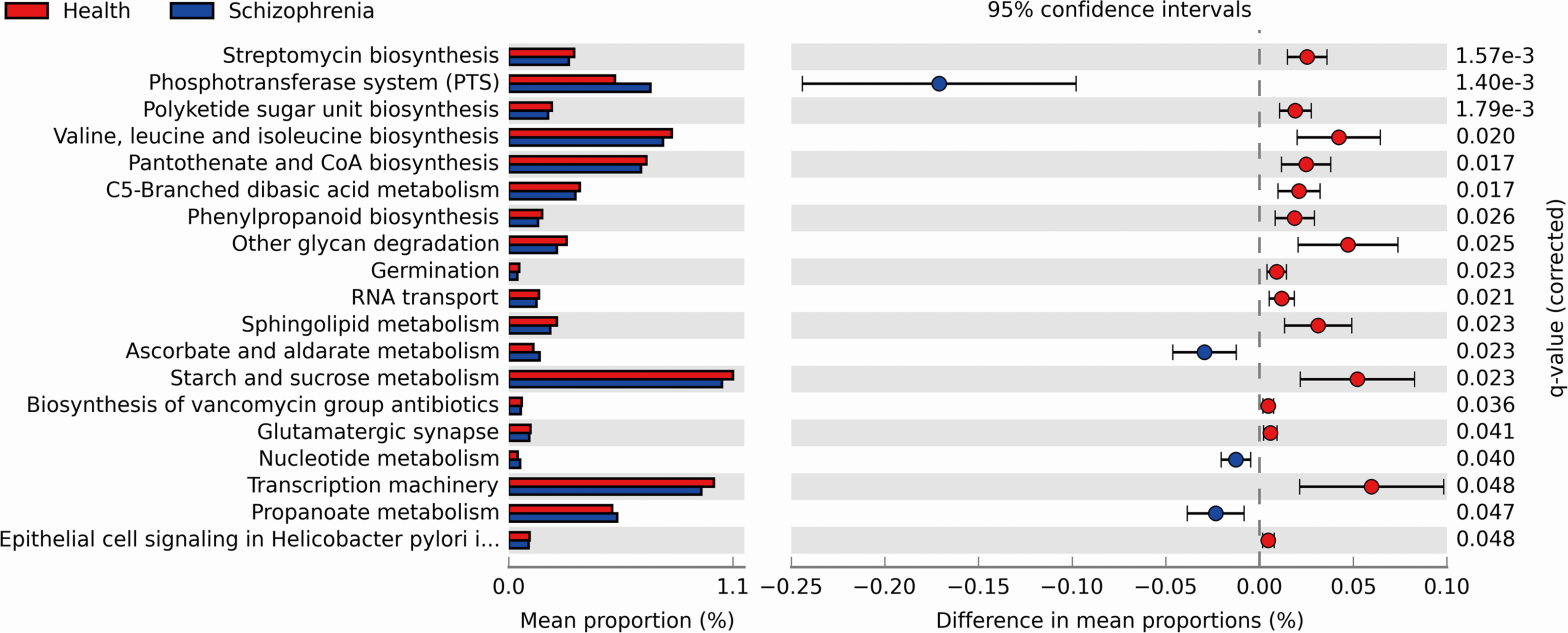
Functional prediction analysis of two groups using PICRUSt. In the figure, the abundance of the biological pathways between the two groups are statistically significant (*p* < 0.05, FDR corrected). Red and blue represent the NC group and the SZ group, respectively.

We performed PICRUSt analysis to predict the genetic potentials of the fecal microbiota metagenome based on 16S rRNA sequences. PICRUSt predicted metagenome content to Level 3 KOs and identified 328 functional pathways belonging to different Level 1 KOs, including 19 Cellular Processes, 28 Environmental Information Processing, 28 Genetic Information Processing, 40 Human Diseases, 146 Metabolism, 40 Organismal Systems and 40 Unclassified pathways (Data S1). We identified 19 significantly different functional pathways (Fig. 4, *p* < 0.05, FDR corrected). We found that varieties of biosynthesis and metabolism pathways were enriched in the NC group, such as Polyketide sugar unit biosynthesis, Valine, Leucine and Isoleucine biosynthesis, Pantothenate and CoA biosynthesis, C5-Branched dibasic acid metabolism and Phenylpropanoid biosynthesis. While Ascorbate and aldarate metabolism, Nucleotide metabolism and Propanoate metabolism pathways were enriched in the SZ group.

### Relationship with clinical characteristics

**Figure 5:**
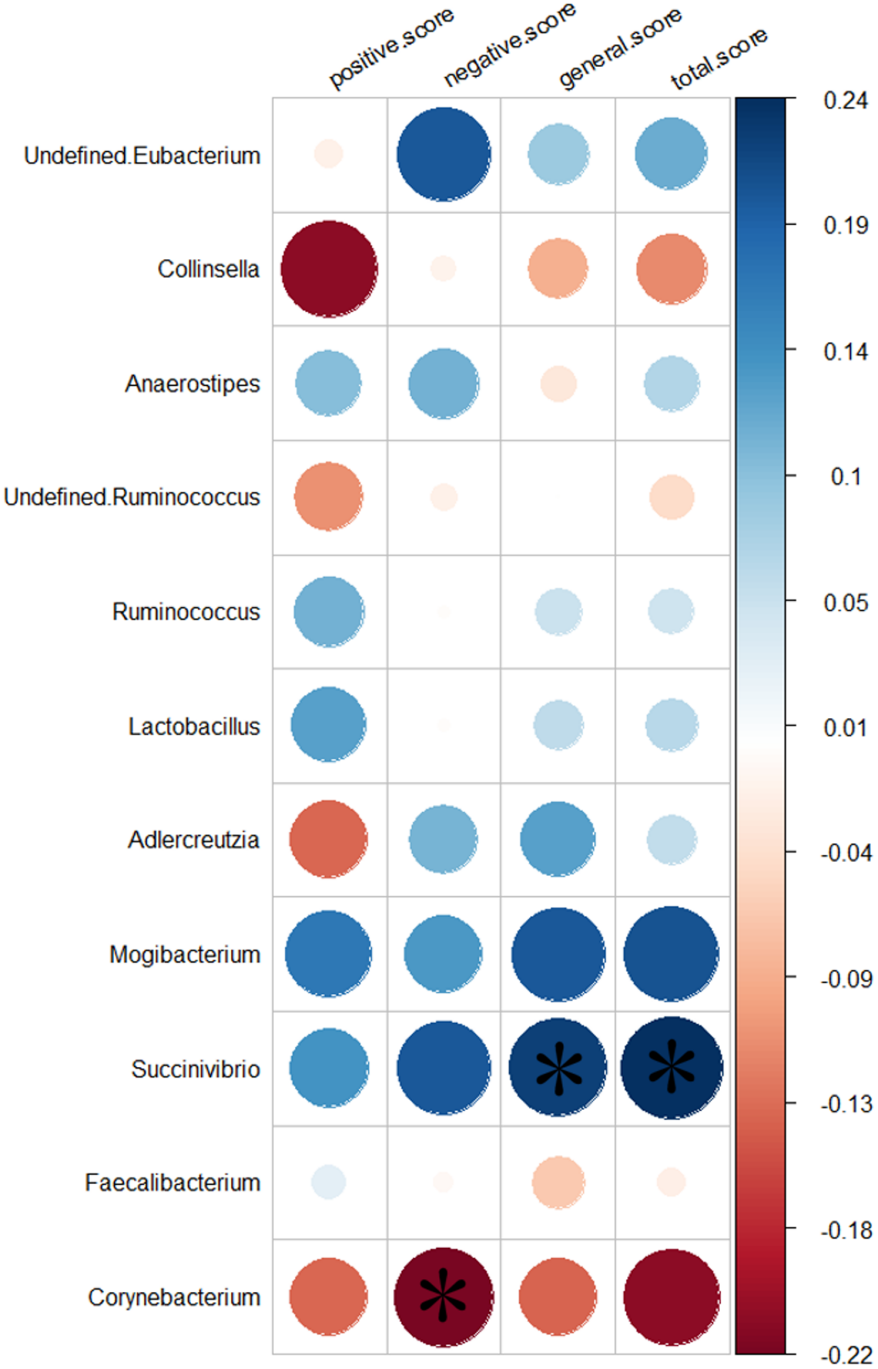
Correlation between the relative abundances of the alter genera and PANSS scores. The color bar indicates the value of Pearson correlation’s coefficient. The size of circles indicates the degree of significance. “*”: *p* < 0.05, uncorrected.

We analyzed the relationship between 11 genera altered in the SZs and the PANSS scores. Greater severity of SZ symptoms was positively correlated with the abundance of the genus *Succinivibrio* (total score, *r* = 0.24, *p* = 0.032, uncorrected; general score, *r* = 0.22, *p* = 0.046, uncorrected). While increased negative symptoms were negatively associated with the abundance of the genus *Corynebacterium* (negative score, *r* = −0.22, *p* = 0.044, uncorrected). The results of the relationship between the 11 genera and the severity of symptoms are shown in Fig. 5.

## Discussion

To the best of our knowledge, this study is the first to indicate that altered gut microbiota is significantly correlated with symptom severity in SZs from South China. Consistent with previous studies, our results demonstrate that the SZs showed altered gut microbiome composition, including two phyla and 11 genera (De Filippo et al., 2010; Huttenhower et al., 2012; Nam et al., 2011; Zhang et al., 2015). Importantly, *Succinivibrio* was more abundant in SZs and correlated positively with the severity of symptoms. In contrast, *Corynebacterium* was more highly represented in SZs and negatively associated with the severity of negative symptoms, which may suggest that a greater abundance of *Corynebacterium* in SZs could remit the symptoms of blunted affect, poverty of speech and loss of drive. Thus, we speculated that an altered gut microbiome profile contributes to the pathogenesis and remission of SZ. Interestingly, *Succinivibrio* was detected in the SZs but not in the NCs, which may further suggest that *Succinivibrio* plays an important role in the development of SZ. In addition, we found that *Lactobacillus* was significantly higher in SZs. Schwarz et al. (2018) found that the abundance of *Lactobacillus* was significantly increased in first episode SZ and was positively correlated with the severity of symptoms. However, the correlation between *Lactobacillus* and symptom severity was not significant in this study. One possible reason was the drug use of the subjects included in this study.

Second-generation antipsychotics (SGAs) have been used successfully for the treatment of SZ (Skonieczna-Żydecka et al., 2019), risperidone (RIS) and olanzapine (OLZ) are the most frequently prescribed atypical SGAs (Hálfdánarson et al., 2017). However, long-term SGA treatment can cause health consequences including significant weight gain and hypertriglyceridaemia (Chintoh et al., 2009; De Hert et al., 2011; Galling & Correll, 2015). In this study, 91% SZs was treated with antipsychotics (Data S2). Our results showed that BMI and TG of SZs were significantly higher than that Of NCs, which was consistent with previous studies. Skonieczna-Żydecka et al. (2019) concluded that metabolic disturbances during SGA treatment may be the consequence, at least in part, of gut dysbiosis. In addition, we were surprised to find that the TC in the NC group was significantly higher than that in the SZ group, which we speculated might be due to the higher alcohol intake ratio in the NC group (30 vs. 3, *p* = 3.36 × 10^−8^, uncorrected). An expanding body of evidence supports the notion that microbes can metabolise drugs and vice versa drugs can modify the gut microbiota composition. Bahr et al. (2015) identified the *Bacteroidetes*/*Firmicutes* ratio was significantly lowered in chronic and short-term RIS users. Morgan et al. (2014) revealed decreased alpha diversity, lower abundance of class *Bacteroidia*, and increased abundances of *Erysipelotrichia*, *Actinobacteria* and *Gammaproteobacteria* in female mice treated with OLZ. However, Kao et al. (2018) demonstrated no significant effects of OLZ on gut microbiota in female rats. Pełka-Wysiecka et al. (2019) further explored the gut microbiota and OLZ treatment interactions, they classified the included SZs as responders and non-responders, there were no differences in gut microbiota compositions at phyla and genus levels. Hence, the effect of antipsychotics between gut microbiota needs further study.

There are a number of bidirectional signaling pathways by which the gut microbiota, acting via the brain-gut axis, can impact the brain (Kelly et al., 2017), including amino acid metabolism (Saleem et al., 2017), immune system modulation (Erny et al., 2015), hypothalamic-pituitary-adrenal (HPA) axis (Mudd et al., 2017), vagus nerve (Bravo et al., 2011) and the production of bacterial metabolites, such as short-chain fatty acids (SCFA) (Tan et al., 2014). In this study, PICRUSt results showed multiple SCFAs and amino acid metabolic pathways that were significantly enriched between the two groups (Fig. S1). SCFAs are the main metabolites of the gut microbiota (Wong et al., 2006); SCFAs can enter the central nervous system through the blood-brain barrier (De Vadder et al., 2014), stimulating TNF in the body (Morris et al., 2017), activating microglia (Sampson et al., 2016), interfering with membrane metabolism of cells, and thus may induce SZ. He et al. (2018) reported that an increased relative abundance of *Lactobacillus* in SZs can stimulate TNF production. Based on this, it is speculated that the increased *Lactobacillus* may induce changes in inflammatory factors and induce SZ. Amino acids and derivatives participate in the biosynthesis and downstream effects of numerous neurotransmitters (Cao et al., 2018). We found that the tryptophan metabolism was significantly enriched in the fecal microbiome of SZs. Zhu et al. (2019) reported that the tryptophan level in mice transplanted with SZ fecal microbiota was significantly lower than that in NC mice, and they also found that tryptophan biosynthesis was significantly enriched in the fecal microbiome of NC mice by shot-gun metagenomic sequencing. Tryptophan is an important source of 5-hydroxytryptamine (5-HT). Tryptophan and kynurenine can cross the blood-brain barrier and have a significant effect on the metabolism of neurotransmitters (Agus, Planchais & Sokol, 2018). Above all, these investigations suggested that gut microbiota may profoundly affect the amino acid metabolism pathway and neurotransmitter levels in SZ patients.

Several methodological issues need to be addressed. First, we did not control the effect of antipsychotic therapy on the gut microbiota due to the lack of data. The form of clinical information will be modified and the data of antipsychotic will be collected. Besides, we plan to recruit patients of first-episode SZ in the future study. Second, in this preliminary study, we adopted the method of 16S rRNA gene sequencing, which has a low phylogenetic power at the species level. According to the findings of this study, we have selected the specific subjects and have applied the metagenomic analysis in the next study. Third, the BMI of the part of subjects and the diet information of all subjects were lacked in this study. Further investigations will include all these data.

## Conclusions

In conclusion, our findings provide evidence of altered gut microbial composition in patients who have SZ. In addition, we found that *Succinivibrio* and *Corynebacterium* were associated with the severity of symptoms for the first time, which may provide some new biomarkers for the diagnosis of SZ.

## Supplemental Information

10.7717/peerj.9574/supp-1 Supplemental Information 1 Richness and diversity in the NC and SZ groups. Values are shown as mean ± SD.

10.7717/peerj.9574/supp-2 Supplemental Information 2 Function prediction analysis results of 16S rRNA.

10.7717/peerj.9574/supp-3 Supplemental Information 3 Demographic and clinical data.

10.7717/peerj.9574/supp-4 Supplemental Information 4 Sequencing Data.

10.7717/peerj.9574/supp-5 Supplemental Information 5 Feature table with taxanomy.

10.7717/peerj.9574/supp-6 Supplemental Information 6 Metadata: participants’ grouping, gender, age, and years of education.

10.7717/peerj.9574/supp-7 Supplemental Information 7 Functional prediction analysis of two groups using PICRUSt. The abundance of the biological pathways between the two groups are statistically significant (p < 0.05, uncorrected). Red and blue represent NC group and SZ group, respectively.

## Additional Information and Declarations

### Data Availability

The following information was supplied regarding data availability: The sequences are available at NCBI GenBank: MT545156–MT547172 and in the Supplemental File.
