# Supplementary material for: Altered gut microbiota associated with symptom severity in schizophrenia
Source: PeerJ. 2020 Jul 29;8:e9574. doi: 10.7717/peerj.9574 (PMC7395597; doi:10.7717/peerj.9574)
Supplement: Supplemental Information 1 [file peerj-08-9574-s001.docx]

Supplementary Table 1 Richness and diversity in the NC and SZ groups. Values are shown as mean ± SD.

| Characteristic | NC group | SZ group | H | p-value |
| --- | --- | --- | --- | --- |
| Shannon | 4.43±0.76 | 4.34±0.74 | 0.80 | 0.37 |
| Evenness | 0.66±0.09 | 0.65±0.09 | 0.88 | 0.35 |
| Faith’s PD | 8.68±1.97 | 8.83±2.10 | 0.09 | 0.77 |
| Observed OTUs | 106.96±31.35 | 105.79±35.16 | 0.16 | 0.69 |
