# Supplementary figures and images for: Altered gut microbiota associated with symptom severity in schizophrenia
Source: PeerJ. 2020 Jul 29;8:e9574. doi: 10.7717/peerj.9574 (PMC7395597; doi:10.7717/peerj.9574)

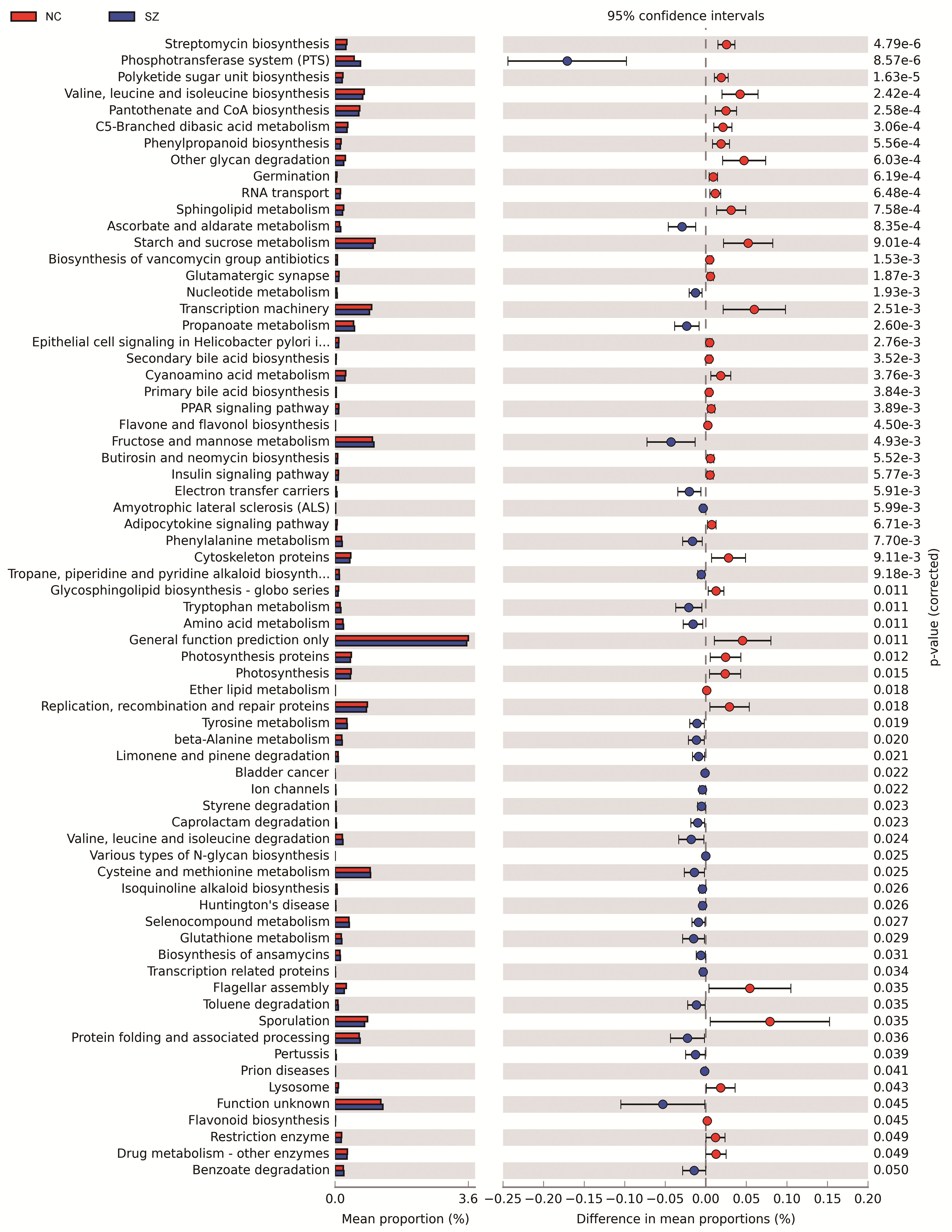

Supplement: Supplemental Information 7 — The abundance of the biological pathways between the two groups are statistically significant (p < 0.05, uncorrected). Red and blue represent NC group and SZ group, respectively. [file peerj-08-9574-s007.png]
